# Characterization of teff straw from selected teff varieties from Ethiopia

**DOI:** 10.1016/j.heliyon.2023.e17422

**Published:** 2023-06-17

**Authors:** Belete Tessema, Girma Gonfa, Sintayehu Mekuria Hailegiorgis, Venkatesa Prabhu Sundramurthy

**Affiliations:** a Department of Chemical Engineering, Addis Ababa Science and Technology University, 16417, Addis Ababa, Ethiopia; b Biotechnology and Bioprocess Center of Excellence, Addis Ababa Science and Technology University, 16417, Addis Ababa, Ethiopia; c Nanotechnology Center of Excellence, Addis Ababa Science and Technology University, 16417, Addis Ababa, Ethiopia

**Keywords:** Teff straw, Proximate analysis, Chemical composition, Silica, Ash content

## Abstract

Utilization of biomass is important both for economic and environmental projection purposes. To use biomass for industrial applications as well as to reduce its pollution load on environment, it is important to characterize and determine the compositions of the biomass. In this work, the proximate and chemical analyses of straws of four (Dagim, Filagot, Kora and Kuncho) Teff (Eragrostis tef) varieties were investigated with three replications. The thermographic and FTIR of the teff straws and the ashes were also studied. The volatile matter contents of the teff straws were 78.80, 77.00, 80.20 and 80.60% for the Dagim, Kuncho, Kora and Filagot varieties, respectively. The ash contents of the straws were 6.34% for Dagim, Kuncho and Kora while the value is 6.00% for Filagot. The fixed carbon contents of the straws were 14.86, 16.67, 13.47 and 13.40% for Dagim, Kuncho, Kora and Filagot varieties, respectively. The silica contents of the teff straw for the Filagot, Kora, Dagim, and Kuncho varieties are 5.92, 5.66, 4.94, and 4.70%, respectively. This corresponds to 92.21, 91.59, 77.19 and 87.20% silica contents in the ashes produced from Filagot, Kora, Dagim, and Kuncho varieties, respectively. The results show that the proximate and chemical composition of ash produced from teff straws show slight differences. Moreover, the silica content of the teff straw is comparable with the values reported for rice husk and wheat straw. Thus, teff straw can be used for the production of silica.

## 1 Introduction

Today the major challenges facing the globe are the fast diminishing of natural resources and the generation of excess wastes to the environment [1]. Biomass waste is one of the major wastes generated and damped into the environment annually and its generation has been increasing enormously [2]. Utilization of biowastes is important both for economic and environmental protection purposes [1,3]. Hence, various national and regional bioeconomy strategies have been developed and attracted attention both from academicians and industries. Bioeconomy can be defined as “an economy where the basic building blocks for materials, chemicals, and energy are derived from renewable biological resources [4]. One of the applications of biomass wastes is its use for the recovery of important biobased materials, such as silica [5,6]. Silica has numerous industrial applications, such as glass [7]**,** cement [8]**,** supercapacitors [9], batteries [10], pharmaceuticals and cosmetics [11], etc. Various biomass wastes have been investigated for their contents of silica and extraction of silica from the wastes. For instance, rice husk comprises 70–80% organic substances and the remaining 20–30% constitutes mineralogical components such as silica, alkalis and trace elements [5]. Other biomass wastes, such as wheat straw [12], maize stalk [13], teff straw [14], sugarcane bagasse [15], oat husk [16], cassava periderm [15] and teff straw [14] been investigated for extraction of silica. Previous studies indicate the silica content of the biomass waste depends on the nature of the biomass [[17], [18], [19]] and varieties of the biomass its growth geographical locations [6,20]. Therefore, it is important to investigate the proximate and ultimate composition of the biomass to use the biomass for extraction of silica.

In the current work, we characterized the ash produced from teff husk of four varieties from Ethiopia. Teff (Eragrostis tef) is an indigenous and one of the major cereal crops in Ethiopia [21]. In 2012, teff shares about 22.6% (about 3,098,887 ha) of arable land, and it is the third (after maize and wheat) crop in terms of annual cereal crop production in Ethiopia [22,23]. The crop generates about 5000 kg/ha of straw which makes an annual teff straw generation of 15.5 million tonnes/year [23]. Teff is a fine-stemmed and tufted annual crop characterized by a large crown, a shallow diverse root, and many shoots system [21]. There are more than 40 teff varieties in Ethiopia [24]. Every year, large quantities of teff straw are discarded like garbage in Ethiopia [25]. Teff straw does not have significant commercial value in Ethiopia. However, there are traditional usage, such as feeding animal, mud mix for houses and mostly in rural areas. In most cases, it is subjected to open-air burning for the release of nutrients followed by cultivation, which might result in air pollution [14,26]. Currently, some studies have been investigated to utilize or commercialize this biomass waste (teff straw) for feed livestock [27], adsorption of pollutants, such as heavy metals from water [28,29], production of bioethanol [30], it can be a potential lignocellulosic biomass for the production of biofuels and reducing sugar [31], isolation of cellulose fibrils [32] and possibility of reinforcement in polymer composites for lightweight applications [33]*.* Moreover, it has been also studied for extraction of valuable materials such as silica [14,34,35]. The results show that the teff straw ash contains up to 91.81% silica (SiO_2_) which is the highest next to the value reported for sorghum bagasse (96.36%) [36], rice husks of (92.9 and 94.79%) [37,38], maize leaves (93.0%) [13] and wheat husk (92.30%) [38]. However, as it was observed for many biomass, including rice husk, the ash contents as well as silica contents of the biomass varieties and their geographical locations [20,39]. This could be true for teff straw as several teff varieties grow in various ranges of geographical locations and environmental conditions.

Teff is the primary cereal crop in Ethiopia, where it is farmed annually by over 6.5 million smallholder farmers on about 30% of the total land designated for cereal crops [24]. The majority of the straw made from teff is available as junk. Currently, it is employed as animal feed and blended with mud to construct traditional homes without commercial use. A thorough search of the literature reveals that no studies and characterized for silica content as well as for thermal property determination from varieties of teff straws such as Dagim, Kuncho, Kora and Filagot. Characterizing these abundantly available varieties agricultural waste material in to useable product is worthy economically and pollution prevention environmentally. Therefore, in this work, we investigated the effect of teff varieties on the physical and chemical properties of the resulting ash and their silica contents. These teff varieties, were collected from the local agricultural research institute and analyzed for various thermophysical properties and compositional analyses. Proximate analyses and chemical analyses were performed to determine the composition of the biomass and its silica contents. Thermogravimetric analyses and Fourier transform infrared spectroscopy analyses were performed to study the thermal properties of the biomass and their Fourier transforms infrared functional groups.

## 2 Materials and methods

### 2.1 Sample collection and preparation

The four teff varieties (Dagim, Kuncho, Kora and Filagot) were obtained from Debre Zeit Research Centre, Ethiopia. First, impurities such as leaves, sands, soil and clays were removed from the teff straw. Then, it was soaked in tape water for 24 h and then washed five times using tap water to remove dust and soil mud. Then, it was dried in sunlight and grounded into power and stored in air-tight polyethylene bags.

### 2.2 Proximate analysis

The proximate analyses were carryout to determine moisture content, volatile matter content, ash content, and fixed carbon content. The average value from each experiment which carried out in triplicate has been reported in this study. The moisture content was determined using ASTM-D 3173. Teff straw powder samples (5 g) were placed in small aluminium evaporating dishes and placed in an air-forced drying oven (NÜVE MF 106). The samples were dried for 24 h in the oven keeping the oven temperature at 105 °C. The moisture contents were determined using equation (1).

(1) $$MC\;\left(\%\right)=\frac{m_{1}-m_{2}}{m_{1}}$$

where, *m*_*1*_ is the initial weight of the sample, *m*_*2*_ is the weight of the dried sample and *MC* (%) is the moisture content in percentage.

The volatile matter contents were determined using ASTM D-3175. A known mass of grounded teff straw sample in a crucible and placed a muffle furnace (FE-37-FA-013) at a temperature of 950 °C for 7 min. The volatile matter percentage was determined using equation (2).

(2) $$VM\;\left(\%\right)=\frac{m_{3}-m_{4}}{m_{3}}$$

where, where, *m*_*3*_ is the initial weight of the sample, *m*_*4*_ is the weight of the sample after treating in the furnace, and *MV* (%) is the volatile matter content in percentage. The ash content (AC) was determined by heating a known mass of the grounded teff straw sample in the muffle furnace (FE-37-FA-013) at 650 °C for 3 h using ASTM D-3174. The ash content was determined using equation (3).

(3) $$AC\;\left(\%\right)=\frac{m_{5}-m_{6}}{m_{5}}$$

where, where, *m*_*5*_ is the initial weight of the sample, *m*_*6*_ is the weight of the sample after treating in the furnace, and *AC* (%) is the ash content in percentage. Finally, the foxed carbon contents were determined using equation (4).

(4) FC(%)=100−(MC+VM+AC)

where, *FC* is the fixed carbon (%), *MC* is the moisture content (%), *VM* is the volatile matter (%), and AC is the ash content (%).

The term standard deviation refers to a measurement of the data's dispersion from the mean. A low standard deviation implies that the data are grouped around the mean, whereas a large standard deviation shows that the data were more dispersed [40]. Therefore, the proximate analysis of moisture content, volatile matter, ash content and fixed carbon content of the standard deviation.

### 2.3 Chemical composition

The chemical analyses were performed to determine the composition of the teff husks. The compositions of the ashes were carried out using atomic absorption spectroscopy (CB-AAS-3510). The samples were fused in LiBO_2_ and dissolved in HF and analyzed by atomic absorption spectroscopy. The composition of major constituents of SiO_2_ and minor oxides such as Fe_2_O_3_, CaO, K_2_O, P_2_O_5_, Al_2_O_3_, Na_2_O, TiO_2_, MgO and H_2_O was determined by using atomic absorption spectroscopy.

### 2.4 Thermogravimetric and FTIR analyses

Thermogravimetric analysis (TGA) was performed using a thermographic analyser (SDT Q600). The powdered teff straw sample from polyethylene bag was placed within a tiny platinum crucible that is hung from the arm of a microbalance. The complete setup is housed in a tiny oven, whose temperature is controlled and observed [41]. The sample was heated in a given environment air at controlled rate of 20 °C/min. The change in the weight of the substance was recorded as a function of temperature. The temperature was increased at a constant rate for a known initial weight of 10 mg the sample was placed into an alumina crucible and heated from room temperature to 900 °C and the changes in weights were recorded as a function of temperature in °C. This plot of weight change against temperature was recorded for thermogravimetric curve or thermogram; this is the basic principle of TGA. The functional groups of the raw teff straw were analyzed using Attenuated total reflection (ATR) with Fourier Transforms Infrared spectroscopy (Thermo scientific ATR-FTIR, IS50) The Fourier Transforms Infrared (FTIR) spectrum was recorded over a wavenumber of 400 and 4000 cm^−1^. The prepared powder of teff straw from a polyethylene bag, are placed on the ATR crystal and pressed down using a swivel press to provide optimum contact between sample and crystal.

## 3 Results and discussions

### 3.1 Proximate analysis

#### 3.1.1 Moisture content

The moisture contents of the grounded teff straw powder for the four varieties are shown in Table 1. The moisture contents of the current teff straw vary from 7.40% to 9.20%. The current moisture content is in good agreement with the value reported from teff straw [25,34,42]. It can be also noted that the moisture content of the teff straw depends on the variety of the teff. These results are quite close to the values reported for other dried biomasses which vary from 6.00 to 9.38 as indicated in Table 1. The moisture content of teff straw is not extreme and hence can be explored for various applications. Crop residue moisture contents generally vary from 3% to 14% [20]. The moisture content of the biomass depends on the nature of the biomass as well as the methods of drying and drying parameters (drying temperature and temperature) used. The standard deviation of the moisture content of Kora > Kuncho > Dagim > Filagot. Decreasing standard deviation which implies that the data are grouped around the mean.

**Table 1:** Moisture contents of dried teff straws and comparison with other common biomasses.

Biomass	Moisture content (wt. %)					References
	S_1_	S_2_	S_3_	Average value	Standard deviation	
Teff straw (Dagim variety)	9.12	9.30	9.18	9.20	0.092	Present study
Teff straw (Kuncho variety)	8.97	9.15	8.87	9.00	0.140	
Teff straw (Kora variety)	7.93	7.60	7.87	7.80	0.176	
Teff straw (Filagot variety)	7.33	7.50	7.37	7.40	0.089	
Teff straw	–	–	–	7.30	–	[34]
Teff straw	–	–	–	6.0	–	[42]
Teff straw	–	–	–	7.5	–	[25]
Sugarcane bagasse	–	–	–	8.37	–	[15]
Cassava periderm	–	–	–	7.34	–	
Maize stalk	–	–	–	6.30	–	
Rice husk	–	–	–	9.38	–	[43]
Wheat husk	–	–	–	6.00	–	[44]
Rye husk	–	–	–	8.00	–	
Softwood	–	–	–	6.00	–	

S_1_, S_2_ and S_3_: for moisture content sample 1, 2 and 3 respectively.

#### 3.1.2 Volatile matter contents

The contents of the volatile matter for the studied teff straws are shown in Table 2. The volatile matter contents vary from 77.00% to 80.60%. The highest value was obtained for the Kora teff variety while the lowest value is for the Kuncho teff variety. The volatile matter of the current teff straw varieties is slightly higher than the value compared with the previously studied teff straw [34]. The teff straws have higher volatile matter contents compared to other biomasses as shown in Table 2. The volatile content of the current teff straw is slightly lower than the values reported for sugarcane bagasse [45] and palm trunk [46] as indicated in Table 2. The volatile matter contents of the biomass can be as high as 86% [20]. The higher volatile content of the teff straw indicates its potential for producing gaseous energies through thermal decomposition processes such as pyrolysis. The standard deviation of the volatile matter of Kuncho > Dagim > Kora > Filagot. Decreasing standard deviation which implies that the data are grouped around the mean.

**Table 2:** Volatile matter contents of dried teff straws and comparison with other common biomasses.

Biomass	Volatile matter content (wt.%)					References
	S_1_	S_2_	S_3_	Average value	Standard deviation	
Dagim	7.73	7.90	7.77	78.80	0.089	Present study
Kuncho	7.43	7.90	7.77	77.00	0.243	
Kora	8.15	8.21	8.25	80.20	0.050	
Filagot	8.58	8.63	8.59	80.60	0.026	
Teff straw	–	–	–	74.70	–	[34]
Rice husk	–	–	–	72.80	–	[47]
Rice straw	–	–	–	74.70	–	
Corncob	–	–	–	78.70	–	
Rice husk	–	–	–	67.90	–	[48]
Cassava Periderm	–	–	–	58.50	–	[15]
Maize Stalk	–	–	–	66.37	–	
Cotton residue	–	–	–	72.80	–	[49]
Waste wood	–	–	–	73.80	–	
Sugarcane bagasse	–	–	–	83.03	–	[45]
Palm trunk	–	–	–	82.60	–	[46]

S_1_, S_2_ and S_3_: for volatile matter sample 1, 2 and 3 respectively.

#### 3.1.3 Ash content

The ash contents of the teff straw of the studied varieties are depicted in Table 3. The ash content values of the current teff straws slightly vary from 6.00 to 6.34%. The ash content of teff straws from different scholars such as 4 [34], 4.6 [42], 5 [28] and 5.88 [32]. Therefore, based on Table 3, the ash content of the current teff straw varieties is the highest value compared with the previously studied teff straw [28,32,34,42,50]. The straw of the studied four teff varieties have almost the same ash contents. The ash content of biomasses generally varies from 2% to 22% [20]. The current results show teff straw has medium ash content compared to most biomasses. Crop residues such as rice husk, rice straw, and sugarcane leaf exhibit higher ash contents which vary from 10% to 22% [20]. The higher ash content of the biomass indicates its potential applications for the recovery of inorganic components such as silica, metal oxides and minerals. The standard deviation of the ash content of Filagot > Kora > Kuncho > Dagim. Decreasing standard deviation which implies that the data are grouped around the mean.

**Table 3:** Ash contents of dried teff straws and comparison with other common biomasses.

Biomass	Ash content (wt.%)					References
	S_1_	S_2_	S_3_	Average value	Standard deviation	
Dagim	6.36	6.33	6.33	6.34	0.017	This work
Kuncho	6.31	6.34	6.36	6.34	0.025	
Kora	6.39	6.31	6.33	6.34	0.042	
Filagot	6.07	5.94	5.99	6.00	0.066	
Teff straw	–	–	–	4.00	–	[34]
Teff straw	–	–	–	4.6	–	[42]
Teff straw	–	–	–	5.0	–	[28]
Teff straw	–	–	–	5.88	–	[32]
Rice husk	–	–	–	17.9 0	–	[47]
Rice straw	–	–	–	10.10	–	
Rice husk	–	–	–	19.60	–	[48]
Cassava Periderm	–	–	–	4.93	–	[15]
Maize Stalk	–	–	–	4.80	–	
Cotton residue	–	–	–	6.20	–	[49]
Waste wood	–	–	–	3.30	–	
Rice husk	–	–	–	11.34	–	[43]

S_1_, S_2_ and S_3_: for ash content sample 1, 2 and 3 respectively.

#### 3.1.4 Fixed carbon content

The fixed carbon contents in the current four varieties of teff straws vary from 13.40% to 16.67%. The fixed carbon content of straws of the current teff varieties is comparable with the value previously reported for teff straws [34]. However, slight variations in fixed carbon content were observed with the teff varieties. The fixed carbon content of teff straw is generally lower than the values reported for most biomasses (Table 4). The fixed carbon content of teff straw is comparable with the value reported for rice husks. Fixed carbon contents of biomasses generally vary from 8% to 31% [20]. The lower fixed carbon of the teff straws may be attributed to its high volatile matter contents as biomass that contains high volatile matter should contain lower fixed carbon contents. The standard deviation of fixed carbon content of Dagim > Kuncho > Filagot > Kora. Decreasing standard deviation which implies that the data are grouped around the mean.

**Table 4:** Fixed carbon contents of dried teff straws and comparison with other common biomasses.

Biomass	Fixed carbon (%)					References
	S_1_	S_2_	S_3_	Average value	Standard deviation	
Dagim	14.67	14.94	14.98	14.86	0.169	Present study
Kuncho	16.53	16.69	16.78	16.67	0.127	
Kora	13.46	13.49	13.47	13.47	0.015	
Filagot	13.43	13.39	13.38	13.40	0.026	
Teff straw	–	–	–	14.00	–	[34]
Rice husk	–	–	–	9.30	–	[47]
Rice straw	–	–	–	15.20	–	
Corncob	–	–	–	16.20	–	
Rice husk	–	–	–	12.50	–	[48]
Sugarcane Bagasse	–	–	–	18.60	–	[15]
Cassava Periderm	–	–	–	29.40	–	
Maize Stalk	–	–	–	22.53	–	
Cotton residue	–	–	–	21.00	–	[49]
Waste wood	–	–	–	22.90	–	

S_1_, S_2_ and S_3_: for fixed carbon content sample 1, 2 and 3 respectively.

### 3.2 Chemical composition

The chemical compositions of ashes from the four varieties of teff straws are shown in Table 5. The loss on ignition represents the composition of the biomass lost in ash formations. The silica contents of the teff straw for the Filagot, Kora, Dagim, and Kuncho varieties are 5.92, 5.66, 4.94, and 4.70%, respectively. This corresponds to 92.21, 91.59, 77.19 and 87.20% silica contents in the ashes produced from Filagot, Kora, Dagim, and Kuncho varieties, respectively. This shows the silicon content of teff straw depends on the varieties of the teff. It can be also noted that teff straws have high silicon content which is comparable with other high silica-containing biomasses such as rice husk and wheat husk. Silica contents of 92.30% and 92.81% were reported for wheat husk and rice husk, respectively [20,51]. Next to silica, the teff straw ashes contain metal oxides such as MgO, CaO which as usually classified as macronutrients. The percentage of metal and alkali species in the ashes of the studied teff straws are 7.79, 8.41, 12.80 and 22.81% for Filagot, Kora, Kuncho and Dagim, respectively. The alkali oxide contents in crop residue ashes generally vary from 5% to 64% [20]. A high concentration of alkali oxides is usually observed in crop residue ashes with high silicon contents. High metal and alkali metal contents do not affect only the concentration of silica it also affects the recovery of silica from the ashes. Processes such as acid or alkali-based hydrolysis or carboxylic groups-based chelation reaction may be used to remove the metal and alkali and purify the silica in the ash [[52], [53], [54]]. These teff straw variants include both inert and partially unburned components. Most of the unburned substance is carbon. The carbon content of the ash and the Loss on Ignition [55] value are equivalent. Depending on the production circumstances, different teff straw varieties contain varying amounts of LOI.

**Table 5:** Composition of ash contents of straws of the studied teff varieties.

	Filagot	Kora	Dagim	Kuncho
Silicon dioxide (SiO_2_)	5.92	5.66	4.94	4.70
Aluminum oxide (Al_2_O_3_)	<0.01	<0.01	<0.01	<0.01
Ferric oxide (Fe_2_O_3_)	<0.01	0.08	0.04	<0.01
Calcium oxide (CaO)	0.24	0.22	1.12	0.32
Magnesium oxide (MgO)	0.18	0.16	0.24	0.26
Sodium oxide (Na_2_O)	<0.01	<0.01	<0.01	<0.01
Potassium oxide (K_2_O)	<0.01	<0.01	<0.01	<0.01
Manganese oxide (MnO)	0.02	<0.01	<0.01	0.02
Di-phosphorus pentoxide (P_2_O_5_)	<0.01	<0.01	<0.01	0.04
Titanium oxide (TiO_2_)	<0.01	<0.01	<0.01	<0.01
Loss on ignition	94.52	94.32	94.61	95.07

### 3.3 Thermogravimetric analysis

**Fig. 1:**
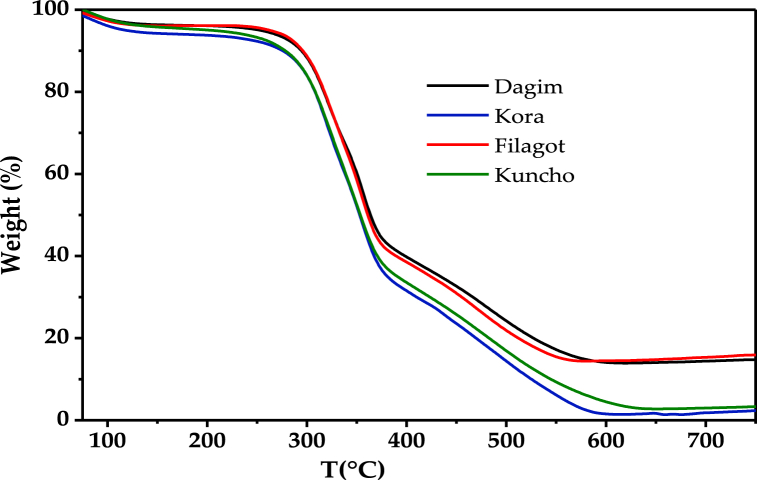
Thermogravimetric graph of teff straw varieties.

The thermogravimetric analysis of the teff straw obtained from the four teff varieties is shown in Fig. 1. The thermogravimetric analysis curve shows three main weight loss regions. The region before 200 °C belongs to dehydration (moisture removal) and the region in the following range up to 400 °C corresponds to volatilization (release of volatile matter). The dehydration stage may be accompanied by the evolution of light volatiles [56]. The region beyond 500 °C is due to carbonization (combustion of combustible materials). The weight loss trend observed for the teff straws is similar to thermogravimetric analyses of most biomasses [15,57].

The weight losses in the first phase were 6.25% for Kora, 5.00% for Kuncho and 4.50% for Filagot and Dagim varieties. High mass losses were observed in the second (volatilization) stage of the thermal decomposition. The weight loss in the second stage for Kora, Kuncho, Filagot, and Dagim are 62.25, 61.50, 57.10, 56.00%, respectively. The teff straw from the four varieties shows similar trends in the thermographic curve although the weight loss at a given temperature varies for the varieties.

### 3.4 Fourier Transforms Infrared spectroscopy

**Fig. 2:**
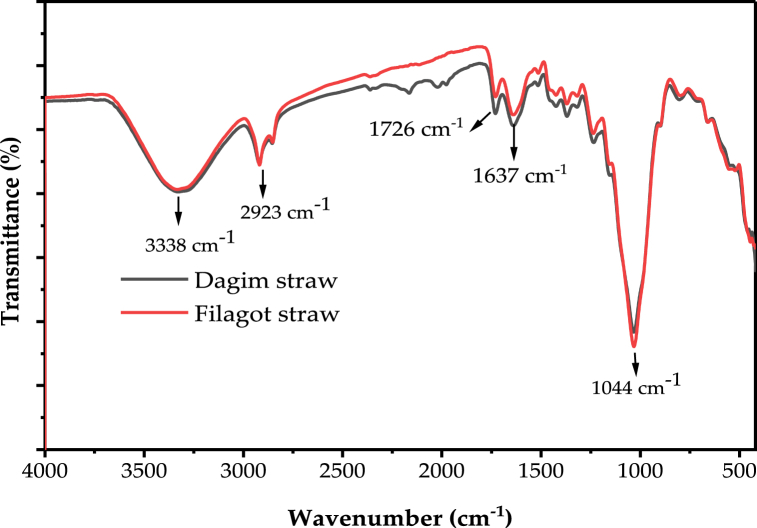
FTIR spectra of teff straw of two teff varieties ((Dagim and Filagot).

**Fig. 3:**
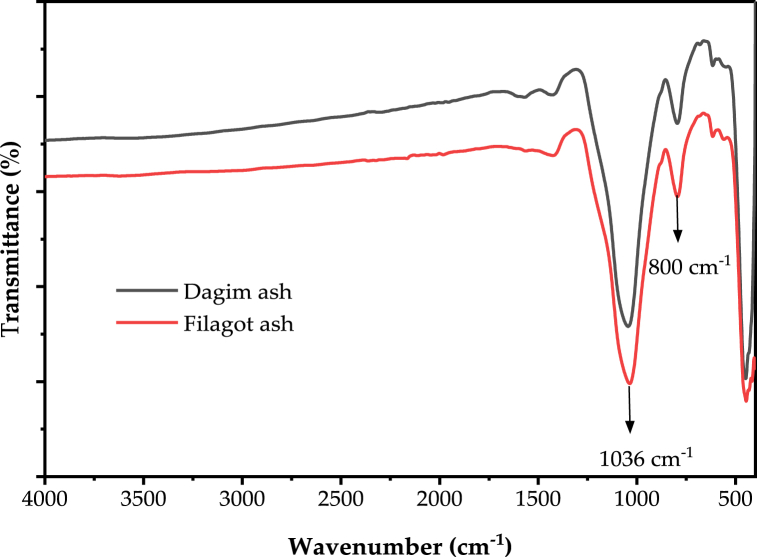
FTIR spectra teff straw ash of two teff varieties ((Dagim and Filagot).

The functional groups of the raw teff straw and resulting ashes were analyzed using FTIR. Different functional groups in teff straw and the ashes were between wavenumber of 400 and 4000 cm^−1^. The FTIR spectrum of the straws and resulting ashes for the two varieties (Dagim and Filagot) are shown in Fig. 2, Fig. 3. The FTIR spectrum of the teff straw is shown in Fig. 2. The peak at a wavenumber of 3380 cm^−1^ corresponds to the OH stretching band in the teff straw. The band at 2923 cm^−1^ indicates the C–H stretching [14] which was observed from unburned carbon impurities residual [58]. The peak found at 1730 cm^−1^ was attributed to carboxylic and carbonyl groups from aldehydes, ketones, as well as aromatic rings, primarily from the lignin component [28]. O–H bond bending vibration was the cause due to the broadband at wave numbers between 1633 and 1645 cm^−1^. The band on wave number from 1036 to 1044 cm^−1^ had been accrued an intense and noticeable absorption peak that was brought on by the asymmetric stretching of the Si–O–Si (siloxane) bonding [59]. While the broad band at 1637 cm^−1^ belongs to H–O–H bending, the broad band between 3000 and 3700 cm^−1^ was attributed to the asymmetric stretching and bending vibrations of silanol - OH groups (Si–O–H) [42].

The FTIR spectrum of the ashes produced from the Dagim and Filagot teff varieties straw is depicted in Fig. 3. The Si–O–Si bonds asymmetric stretching vibration and shear bands are shown by the prominent peaks at 1100 cm^−1^ [58]. The band at 800 cm^−1^ is due to Si due to symmetric stretching vibrations of SiO_4_ [14] or because of the SiO_4_ tetrahedral symmetric stretching vibrations [42]. The peaks observed in Fig. 3 the range of 3750–2500 cm^−1^ for the teff straw disappeared in the resulting ashes. This is because the water in the biomass as well as the organic components that contain hydroxyl functional groups were removed during heat treatments. The FTIR of the current teff straw and resulting ashes are similar to the FTIR spectrum exported by previous authors [14,25].

## 4 Conclusions

In the current work, the proximate and chemical analyses of straws of four variety (Dagim, Filagot, Kora and Kuncho) Teff (Eragrostis tef) varieties were investigated. The ash contents of the straws were 6.34% for Dagim, Kuncho and Kora while the value is 6.00% for Filagot variety. The volatile contents of straws were 78.80, 77.00, 80.20 and 80.60% for the Dagim, Kuncho, Kora and Filagot varieties. The fixed carbon contents of the straws were 14.86, 16.67, 13.47 and 13.40% for Dagim, Kuncho, Kora and Filagot varieties, respectively. The silica contents of the teff straw for the Filagot, Kora, Dagim, and Kuncho varieties are 92.21, 91.59, 77.19 and 87.20%. The results show that the composition of the ash produced from straw of different teff shows slight differences. Moreover, the silica content of the teff straw is comparable with the values reported for rice husk and wheat straw.

## Author contribution statement

Belete Tessema: Performed the experiments; Wrote the paper.

Girma Gonfa: Conceived and designed the experiments; Analyzed and interpreted the data; Contributed reagents, materials, analysis tools or data; Wrote the paper.

Sintayehu Mekuria Hailegiorgis, S. Venkatesa Prabhu: Analyzed and interpreted the data.

## Data availability statement

Data will be made available on request.

## Funding statement

This work was financially supported by Addis Ababa Science and Technology University internal research grant (IGP 006/2023).

## Declaration of competing interest

The authors declare that they have no known competing financial interests or personal relationships that could have appeared to influence the work reported in this paper
